# Antibody-Based Immunotoxins for Colorectal Cancer Therapy

**DOI:** 10.3390/biomedicines9111729

**Published:** 2021-11-19

**Authors:** Laura Sanz, Raquel Ibáñez-Pérez, Patricia Guerrero-Ochoa, Javier Lacadena, Alberto Anel

**Affiliations:** 1 Molecular Immunology Unit, Biomedical Research Institute, Hospital Universitario Puerta de Hierro, 28222 Madrid, Spain; 2 Apoptosis, Immunity and Cancer Group, Aragón Health Research Institute (IIS-Aragón), University of Zaragoza, 50009 Zaragoza, Spain; rakel_albalate@hotmail.com (R.I.-P.); docenteashlaboratorio@gmail.com (P.G.-O.); 3 Department of Biochemistry and Molecular Biology, Faculty of Chemical Sciences, Complutense University, 28040 Madrid, Spain

**Keywords:** immunotoxin, CRC, toxins, monoclonal antibody, toxic payload, targeting moiety

## Abstract

Monoclonal antibodies (mAbs) are included among the treatment options for advanced colorectal cancer (CRC). However, while these mAbs effectively target cancer cells, they may have limited clinical activity. A strategy to improve their therapeutic potential is arming them with a toxic payload. Immunotoxins (ITX) combining the cell-killing ability of a toxin with the specificity of a mAb constitute a promising strategy for CRC therapy. However, several important challenges in optimizing ITX remain, including suboptimal pharmacokinetics and especially the immunogenicity of the toxin moiety. Nonetheless, ongoing research is working to solve these limitations and expand CRC patients’ therapeutic armory. In this review, we provide a comprehensive overview of targets and toxins employed in the design of ITX for CRC and highlight a wide selection of ITX tested in CRC patients as well as preclinical candidates.

## 1. Introduction

Colorectal cancer (CRC) is the third most commonly occurring cancer and the second most lethal, after lung cancer. According to the Global Cancer Observatory, the worldwide number of new cases in 2020 was over 1.9 million (over 3.1 million estimated in 2040), with more than 935,000 deaths, even though in developed countries the death rate has declined in the last decades due to the implementation of screening programs and therapeutic advances. However, the survival rate drops for metastatic CRC and 20% of patients have metastatic disease at diagnosis, while another 25% will develop metachronous metastases [1] (see also the Global Cancer Observatory webpage https://gco.iarc.fr, accessed on 16 November 2021).

For patients with unresectable tumors, radiotherapy and chemotherapy have traditionally been the main strategies for controlling disease, despite their limitations. However, this type of administration of agents with intrinsic toxicity may provoke severe undesirable side effects. Therefore, targeted delivery of cytotoxic agents or toxins is a rational proposal to increase effectiveness and avoid side effects. Targeted therapies, *a priori* more selective and with fewer side effects, include small molecules and monoclonal antibodies (mAbs) directed against tumor-associated antigens (TAA), angiogenic pathways and immune checkpoints. At least nine of these therapies have been approved for the treatment of CRC patients, and numerous agents interfering with different pathways have been brought into clinical trials [2].

In CRC, the epidermal growth factor receptor (EGFR) is the TAA targeted by the two mAbs approved by the FDA, cetuximab and panitumumab. Both are indicated for patients with wild-type KRAS, since mutations in this gene, present in 36% of CRC patients, preclude clinical benefit [3]. In addition, the efficacy of anti-EGFR mAbs is also limited for acquired resistance [4], being the acquisition of mutations that prevent the recognition of a common escape strategy. The anti-vascular endothelial growth factor (VEGF) mAb bevacizumab, the first anti-angiogenic agent in the market, was approved in 2004 for CRC patients, although several trials showed modest improvements in survival. Indeed, angiogenesis inhibition has not fulfilled expectations in cancer patients. Resistance to anti-VEGF therapies has been observed in different cancer types, including CRC, attributed to the activation of alternative signalling pathways. On the other hand, immune checkpoint blockers, which target inhibitory receptors or their ligands and reinvigorate exhausted T cells, have transformed the field of immuno-oncology. The anti-PD1 mAbs pembrolizumab and nivolumab have been FDA-approved for the treatment of mismatch repair (MMR)-deficient CRC patients, who benefit from a response rate of 30–50%; unfortunately, these patients constitute only 4% of the total with metastatic CRC [5].

It is evident that further research is required to develop more effective approaches for metastatic CRC treatment. A promising approach consists in enhancing the clinical activity of anti-TAA mAbs by “arming” them with a potent therapeutic payload. Pharmacological agents, radioisotopes and toxins can all be used as therapeutic moiety in the so-called immune-conjugates, while minimizing off-target effects of the unconjugated agent [6]. Here, we will review the preclinical and clinical development of immunotoxins (ITX) for CRC, defined as immune-conjugates comprising an antibody and a protein toxin (or fragments of them). The term antibody-drug conjugate is sometimes used ambiguously in the literature, but we reserve it for therapeutic agents with small molecule drugs/chemotherapeutic agents as toxic payloads. Advantages of the use of protein toxins are the potency of their catalytic domains, the replication-independent mechanism of action and the escape of common drug resistance mechanisms [7]. Conversely, toxins can be delivered by moieties different from antibodies, such as ligands (cytokines or growth factors), which target the corresponding receptor on the cell surface. For these entities, not included in the scope of this review, alternative denominations have been proposed, such as cytotoxins [8] or ligand-targeted toxins, since key differences exist with canonical ITX [9]. 

In 1978, a seminal paper by Thorpe et al. introduced the concept of using antibodies to redirect the killing activity of toxins [10]. The first ITX were made in the early 1980s, when monoclonal antibodies targeting cancer cells became widely available [11,12]. In 1989, the first recombinant single-chain ITX, produced in *E. coli*, was reported [13]. In 2018, the FDA approved the first antibody-based ITX, moxetumomab pasudotox, for the treatment of hairy cell leukaemia [14], and another one, oportuzumab monatox, was in review as of August 2021 for bladder cancer treatment. Several decades have been necessary, but the therapeutic potential of ITX has been unleashed at last. Important challenges remain, however, for the systemic treatment of solid tumors such as CRC, which are not faced by ITX targeting blood cancers or locally administered. 

## 2. Immunotoxin Development

ITX combine the cell-killing ability of a toxin with the specificity of a monoclonal antibody (mAb), overcoming the limitations of each [15]. Advances in biotechnology have allowed the development of four generations of ITX with improved properties. The first attempts to selectively deliver a toxin to tumor cells were made by chemically coupling a native toxin to a full-length mAb. The toxins were of plant or bacterial origin and mAbs were obtained from mouse hybridomas, so immunogenicity was guaranteed in a non-immunosuppressed host. Linking was produced with reagents that form disulphide bonds connecting the toxin to the mAb. However, due to the presence of several sites for the binding of mAbs and toxins, the obtained products were heterogeneous in their composition and lacked stability, resulting in high variability among batches [16]. Other concerns arose from safety issues, since whole toxins conserved domains implied in non-specific cell entry, that were responsible for some of their side effects, such as vascular leak syndrome (VLS) [17]. 

The ITX design improved considerably with the accumulating information on toxin structures thanks to biochemical and crystallographic studies. In second-generation ITX, the cell-binding domain of the toxin moiety was removed, increasing their tumor specificity [15]. These modified toxins that no longer could bind to normal cells were chemically linked to a full-length mAb moiety, and problems related to costs of production and heterogeneity persisted, although several ITX demonstrated high activity and specificity and were evaluated in phase I trials in cancer patients. Despite showing promising efficacy in vitro, their application in the clinical setting was considerably hampered [18]. Mouse mAbs and non-human toxins gave rise to neutralising human anti-mouse antibodies (HAMA) that rendered treatment ineffective, thus limiting the efficacy of repeated administration of the ITX. Advances in antibody engineering allowed for the chimerization (70% or more human content) and humanization by CDR grafting (90% or more human content) of murine Ab, thus making them less immunogenic, although immune responses were still produced. Moreover, some ITX still bound weakly to normal cells.

**Figure 1:**
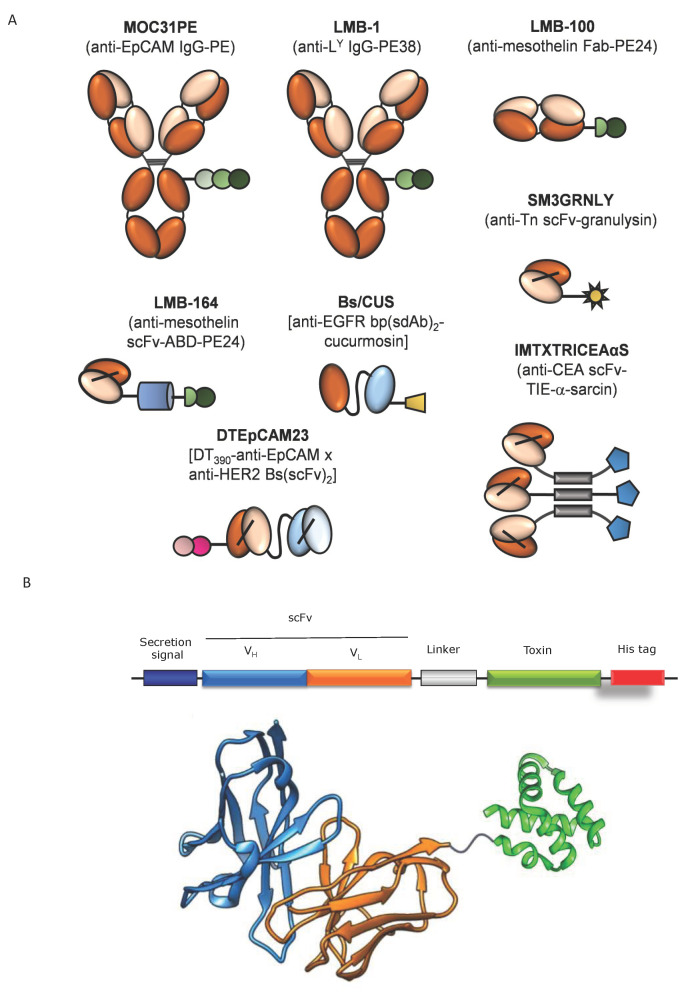
(**A**) Schematic representation of different formats of colorectal cancer-targeted immunotoxins. (**B**) Design and structure of the anti-CEA, granulysin-based immunotoxin MFE23GRNLY. ABD: albumin-binding domain; bs: bispecific; bp: biparatopic; CEA: carcinoembryonic antigen; DT390: truncated version of diphtheria toxin (DT) comprising the first 389 aminoacids; EGFR: epidermal growth factor receptor; EpCAM: epithelial cell adhesion molecule; Fab: antigen-binding fragment; L^Y^: Lewis Y antigen; PE: *Pseudomonas* exotoxin A; PE38: truncated PE (38kDa); PE24: truncated PE (24 kDa); scFv: single-chain Fv; sdAb: single-domain antibody; TIE: collagen trimerization domain; Tn: tumor-associated carbohydrate antigen (α-***O***-GalNAc-Ser/Thr).

Using recombinant DNA technology, the third generation of ITX was developed, overcoming past limitations, and consisting primarily of an antibody fragment genetically fused to a catalytic toxin domain. Application of antibody fragments with the smaller size as targeting moiety improved tumor penetration of ITXs into solid tumors compared to the full-length antibodies [19,20,21]. These constructs are composed of the VH and VL domains, joined together by a 15 amino acid flexible linker in the same polypeptide (scFv, single-chain Fv), or stabilized by an engineered interchain disulphide bond (dsFv). More recently, even smaller single domain Ab or VHH have been used in ITX design, as well as multimeric formats such as diabodies and trimerbodies (see Figure 1). 

Recombinant ITX are homogeneous and less expensive to produce, thanks to the one-step production process. For the recombinant expression of ITX, key points are the choice of the production host and the design of the fusion protein, since ITX may be toxic to the expressing host itself. To avoid or limit the toxicity to productive cells while obtaining a consistent yield in chimeric protein, several systems from bacterial to mammalian host cells have been employed. This topic has been recently reviewed [7]. So far, hundreds of third-generation ITX have been developed, and several PE-based ITX demonstrated promising activity in clinical and pre-clinical studies conducted by the Ira Pastan group. Both moxetumomab pasudotox and oportuzumab monatox belong to this category of recombinant ITX.

While such recombinant ITX showed better efficacy, stability and tumor penetration, immunogenicity still was a major limitation for ITX that used bacterial, plant or fungal toxins, rapidly reducing their clinical success [22]. Different approaches to reduce the immunogenic potential of ITX have been proposed [17,23]. In this context, the use of fourth-generation ITX, also known as human cytolytic fusion proteins (hCFP) or humanized ITX, is one of the approaches that is under ongoing experimentation. In these ITX, the foreign toxins are replaced by cytotoxic human proteins, thus avoiding the immune-mediated reaction against the toxin [24,25,26]. Several human proteins have been used to generate these fourth-generation ITX, including the microtubule-associated protein tau, RNases, granzyme B, death-associated protein kinase [26] and, more recently, granulysin [27,28]. Among those approaches, granzyme B-based ITX have been the most studied and further improved, demonstrating a therapeutic effect in several preclinical models in vivo [24]. 

## 3. Cytotoxic, Non-Human Moieties

### 3.1. Bacterial and Plant Toxins

The bacterial toxins *Pseudomonas* Exotoxin A (PE) and *Diphtheria* toxin (DT) together with the plant ribosome-inactivating proteins (RIP) ricin and aspirin have been most frequently studied for therapeutic purposes but several others are under evaluation, predominantly in the oncological field [10,11,12,13]. Toxins are powerful, natural weapons that have increased in their toxicity by the pressure of natural selection over millions of years and subsequently only a tiny number of molecules is needed to exert overwhelming effects. PE and DT directly inactivate the mammalian elongation factor (EF) by ADP ribosylation, thereby inhibiting amino acid chain elongation during protein synthesis. Ricin, saporin and other RIP, such as pokeweed antiviral protein (PAP), gelonin, bouganin and trichosanthin, depurinate a specific adenine base located in the universally conserved GAGA-tetraloop present in ribosomal RNA [29,30,31]. The final effect is a consequence of the irreversible blocking of protein synthesis, which in turn causes cell death. While type II RIPs are formed by two domains, a catalytic and a binding subunit, type I RIPs have only the catalytic subunit. As a result, the latter have a cytotoxic efficacy that is a hundred-fold lower. It was soon clear that the binding domain of the type II RIP was hazardous and should be inactivated or eliminated. In fact, the cell-binding moiety of ricin, the B chain, is a lectin that is able to recognize galactose residues, driving the catalytic active A domain inside virtually every type of cell. For this reason, the B domain was replaced by the corresponding antibody-based targeting moiety in ricin-based ITX [32]. Similarly, truncated forms of PE of 38kDa or 40kDa (named PE38 and PE40, respectively), deprived of the natural targeting moiety, have been widely used in ITX design [13].

### 3.2. Fungal Ribotoxins

Ribotoxins are RNases that belong to the fungal extracellular RNase family. They have emerged as promising candidates for targeted cancer therapies. Among them, α-sarcin stands out as the most representative member. Their cytotoxic character is due to their ability to interact with acidic phospholipids, which are particularly abundant in the tumor cell membranes, allowing them to internalize to the cytosol where they exert their ribonucleolytic activity. Ribotoxins exert their specific activity on the same target as RIP, the sarcin–ricin loop (SRL), causing a single cleavage of rRNA that leads to ribosome inactivation and consequently protein biosynthesis inhibition, resulting in cell death by apoptosis. Together with their potent and specific ribonucleolytic activity, their small size, high protease resistance, high thermo-stability and low immunogenicity make them toxins with a high potential for their inclusion in antibody-based targeted therapies [33,34]. 

## 4. Human Cytotoxic Proteins

### 4.1. RNases

RNases constitute a highly heterogeneous family of enzymes that hydrolyse phospodiester bonds present in all RNAs. Their wide range of biological activities has been further described, with some of them exhibiting intrinsic preferences from tumor cells. Moreover, their fusion to a cell-targeting antibody has demonstrated enhanced antitumor specificity and efficiency, such as against mesothelioma, melanoma, or pancreatic cancer [35,36,37]. In this sense, human RNases belonging to the RNase A superfamily, such as HP-RNase (RNase 1), human eosinophil-derived RNase (EDN, RNase 2) or angiogenin (Ang, RNase 5), have been used in the design of humanized ITX or hCFP. They exhibit quite different mechanisms of action, including double-stranded RNA (dsRNA) hydrolysis, cleavage of RNAs at the end of pyrimidine bases or degradation of tRNA located in exposed anti-codon, respectively.

HP-RNase-based ITX have been described as targeting breast cancer or lymphoma, among others [38,39], and displaying high antitumor efficacy both in vitro and in vivo. EDN fused to an scFv anti-TFR (CD71) exhibited selective in vitro cytotoxicity in leukaemia, melanoma or renal carcinoma cell lines [40]. Similar results have been obtained with Ang targeting EGFR^+^ [41], CD22^+^ [42] or CD64^+^ cells [43]. However, their clinical translation has been delayed due to the presence of the endogenous ribonuclease inhibitor (RI), which reduces their efficacy. Human RNases are tightly regulated by RI by the formation of an irreversible and enzymatically inactive complex with monomeric RNases [37]. Different strategies have been developed to avoid RI-mediated inhibition. Dimerization or multimerization of RNases fused to Ab prevents RNase inactivation [44]. Alternatively, the inclusion of mutations that decrease the affinity of the inhibitor in the QBI-139 variant of angiogenin has significantly increased its antitumor efficacy against solid tumours in a phase I trial [45]. More examples of antibody fusion proteins with human RNases are revised in [46].

### 4.2. Microtubule-Associated Protein Tau (MAP-Tau) 

MAP tau belongs to the family of microtubule-associated proteins (MAP) which are involved in microtubule stability modulation, resulting in cell cycle arrest [47]. For this reason, they have been used to generate targeted ITX [26]. The first MAP tau-based ITX was obtained using an activated MAP tau fused to an scFv anti-EGFR [48]. Later on, several pre-clinical studies were developed against different solid tumors by fusing MAP tau to different scFvs, targeting lymphoma, pancreas carcinoma or prostate carcinoma, among others [49,50].

In spite of the promising results, the therapeutic efficacy of MAP tau-based ITX must be further enhanced. The intracellular pathway followed by MAP tau seems to result in an inefficient release from endosomes, causing changes in protein conformation [49]. Further investigations must be undertaken to clarify the mechanism of release to the cytosol, also considering the addition of processing signal sequences, in order to improve the cytotoxic activity of MAP tau-based constructs [26,50].

### 4.3. Granzyme B

Granzyme B is a highly cytotoxic molecule present in the granules of cytotoxic T lymphocytes (CTL) and NK cells and is crucial for immune-mediated defense against virus infection and cancer development [51,52]. Granzyme B is a serine-protease with specificity for Asp residues, whose optimal pH for enzymatic activity is 7.4 [53]. In CTL and NK cells, granzyme B is stored together with perforin, other granzymes and granulysin, inside cytotoxic granules of lysosomal nature, in which pH is acid and granzyme B is inactive [54]. Thus, for granzyme B to exert its cytotoxic activity, it needs to be localized in the cytoplasm of target cells. The granzyme B delivery inside the cytoplasm of target cells is mediated by the pore-forming protein perforin [55]. If granzyme B is just endocytosed by the cells in the absence of perforin, it follows the endosomal route and does not exert cytotoxic activity [56]. Hence, for granzyme-B based ITX to be active, they need to be combined with endosomolytic agents such as chloroquine [57]. 

### 4.4. Granulysin

Granulysin is another protein present in the cytotoxic granules of human CTL and NK cells [58]. Although its main function is antimicrobial, acting directly on bacteria and parasites [59,60], it has been demonstrated that recombinant granulysin is also able to induce the apoptotic death of tumor cells [61,62,63]. The granulysin mechanism of action is initiated by the interaction of the positively charged granulysin with negatively charged membrane phospholipids [64]. This interaction is dependent on the net negative charge of the membrane and is reduced when the cholesterol/phospholipid ratio increases [65]. This explains why granulysin is especially active on bacterial lipid membranes, devoid of cholesterol, and less active on eukaryotic lipid membranes [65]. Although granulysin, contrary to perforin, is not able to induce the formation of pores in the membrane, this interaction provokes an alteration of the membrane structure enough to alter the ionic equilibrium [64] and increase the cytoplasmic Ca^2+^ concentration [61,66]. This increase in the cytoplasmic Ca^2+^ concentration then causes the generation of mitochondrial ROS and, together with the action of the pro-apoptotic member of the Bcl-2 family Bim, initiates the mitochondrial apoptotic pathway [61,67,68]. This membrane-based activity of granulysin and granulysin-based ITX constitutes a new ITX mechanism of action, non-dependent on internalization and release from the endosome. The efficacy of the intra-tumor injection of recombinant granulysin was demonstrated in several in vivo models of tumor development in athymic mice. Interestingly, the antitumor activity of granulysin was associated with a massive NK cell infiltration, suggesting a possible immunogenic effect of granulysin-induced tumor cell death [69,70]. In addition, no side effects of this type of treatment were detected in the athymic mice used in these experiments [69].

## 5. Targets in CRC

### 5.1. LewisY

Tumor progression is frequently associated with aberrant glycosylation of cell surface proteins [71]. Lewis^y^ is a difucosylated oligosaccharide, which can be considered a tumor-associated carbohydrate antigen (TACA). Lewis^y^ decors glycoproteins in 60–90% of epithelial cancers, including breast, lung, colon, pancreas, prostate and ovarian cancers [72]. Importantly, Lewis^y^ expression is associated with the clinical stage and the progression of tumors and has been considered a negative prognostic factor, since its overexpression promotes cell adhesion, proliferation, migration and resistance to chemotherapy [73]. Immunohistochemical analysis has demonstrated some expression of Le^y^ antigen in normal epithelial tissues (including the gastrointestinal tract), but it was restricted to secretory borders [74], suggesting that it should be relatively inaccessible to a systemically administered antibody and making it an attractive target for Ab-based therapeutics [75], including T cell-engaging bispecific antibodies [76]. 

### 5.2. CEA

The carcinoembryonic antigen (CEA), also known as CEA-related cell adhesion molecule 5 (CEACAM5), was described in CRC as one of the first identified tumor-associated antigens [77,78], although it is also expressed at lower levels in normal tissues. The heterogeneous CEACAM family belongs to the immunoglobulin superfamily and comprises 12 genes in humans [79,80]. The functions of this family of proteins are very diverse, including modulation of tumor growth and metastasis, innate and adaptive immunity and host–microbial interaction [79,80,81]. CEA is a GPI-anchored molecule that can be released from the cell surface by proteolytic and non-proteolytic mechanisms [82]. CEA serum levels have been used for CRC screening, as well as in predicting prognosis and monitoring responses to treatment [83]. Soluble CEA could bind and block CEA-targeted therapies, but this deleterious effect has not been observed with bispecific anti-CEA x anti-CD3 antibodies [84] or CAR-T cells directed against CEA^+^ tumor cells [85]. 

### 5.3. EpCAM

EpCAM (CD326) was described more than 40 years ago as a CRC-specific antigen recognized by a mAb obtained from mice immunized with human CRC cell lines [86]. Ten years later, the sequence encoding this single-pass type I membrane glycoprotein was published [87]. In 1995, the anti-EpCAM mAb reported by Herlyn et al. was the first ever approved for the treatment of oncologic patients (1995, Germany) under the name edrecolomab, although it was subsequently withdrawn when larger studies showed no benefit compared with standard chemotherapy in CRC [88]. 

As with other TAA, EpCAM is also expressed in normal tissues where it functions as a homophilic cell adhesion molecule, with a role in epithelium integrity. In cancer, EpCAM is regarded as a multifaceted protein involved in the regulation of cell adhesion, proliferation, migration, stemness and epithelial to mesenchymal transition. (EMT) EpCAM is highly overexpressed in a variety of carcinomas beyond CRC. Indeed, the ITX oportuzumab monatox constituted by a humanized anti-EPCAM scFv fused to PE is intended for the intravesical treatment of bladder cancer. Furthermore, the only FDA-approved technology for circulating tumor cell isolation (in colon, breast and prostate tumors), with prognostic potential, is based on the selection of EpCAM-positive cells [89]. 

Overall, EpCAM is an appealing target, although its expression on healthy epithelia has limited the therapeutic window of systemic EpCAM-directed therapies due to on-target/off tumor side effects.

### 5.4. Muc-1/Tn 

The Tn antigen is formed by a *N*-acetylgalactosamine (GalNAc) residue linked to either serine or threonine residues by an alpha-O-glycosidic bond, normally in the context of the membrane glycoprotein MUC1 [90]. Incomplete MUC1 glycosylation in tumor cells leads to the exposure of peptide epitopes, which are masked in healthy cells. The expression of the Tn antigen results from genetic changes that lead to the decreased expression of Cosmc and/or T synthase [91]. In the endoplasmic reticulum, Cosmc binds to the newly synthesized Tn-synthase and it intervenes in the folding of the enzyme and its acquisition of activity. Dimeric T-synthase can then move to the Golgi apparatus, where it exerts its function to add residues of galactose from the donor UDP-Gal to the O-glycan precursor. Normal O-glycans can be sialylated to generate a sialylated 1 O-glycan nucleus or can be expanded to form a nucleus 2 O-glycan. In the absence of Cosmc, a defective folding of T-synthase occurs, and it is retrotranslocated to the cytoplasm, ubiquitinated and degraded in the 26S proteasome. This leads finally to the expression of the aberrant Tn and sialyl-Tn antigens on the cell surface [92]. The Tn antigen has been reported to be present at high levels; in 90% of breast cancers and in 70–90% of colon, lung, cervix, ovary, stomach and prostate tumors, while in normal tissues it is absent or expressed at low levels, constituting one of the TAA selected by the US National Cancer Institute [90,93]. Moreover, it is a target for the development of CAR T cells against solid tumors [94].

### 5.5. GPA33

Glycoprotein A33 (GPA33) is a cell surface antigen overexpressed across a panel of primary and metastatic CRC [95,96], with quite a heterogeneous expression in normal epithelia of the lower gastrointestinal tract [97]. GPA33 belongs to the immunoglobulin superfamily related to junctional adhesion molecules (JAM), with roles in cell adhesion and proliferation [98]. GPA33 is involved in the development and maintenance of the small intestine mucosa during embryonic development [99], showing a drastic decrease of its expression and migration towards the basolateral region in subsequent stages. Such polarisation is maintained in CRC but with increased expression of up to 800,000 molecules/cell [100,101]. GPA33 is rapidly internalised after Ab binding and the subsequent formation of the Ag-Ab complex [102].

GPA33 is, therefore, postulated as a promising candidate for Ab-targeted therapies and ITX design. A fully humanised anti-GPA33 Ab has been reported [103], conjugated with different radioactive isotopes [104,105] and tested in clinical trials [106]. The corresponding scFv (scFvA33) has also been combined with different therapeutic agents [107,108] or included in bispecific Ab designs such as the dual-affinity re-targeting antibody (DART) format [109].

### 5.6. EGFR

EGFR, also referred to as ErbB1, is a tyrosine kinase receptor belonging to a family of four receptors: ErbB1 (EGFR), ErbB2 (HER2), ErbB3 (HER3) and ErbB4 (HER4). They are trans-membrane glycoproteins that are activated by homo- or heterodimerisation upon the binding of their ligand, EGF or TGF-α [110]. Their typical structure consists of an extracellular ligand-binding domain, a trans-membrane domain and an intracellular domain with distinct tyrosine residues that are phosphorylated upon activation. In turn, this activates RAS/RAF/MEK/ERK, PI3K/AKT and JAK/STAT3 signalling pathways and plays an important role in the regulation of growth, survival and differentiation of a large number of different cell types [111,112].

Due to this central role in proliferation, survival and angiogenesis, EGFR overexpression confers a major advantage to tumour cells. Specifically, EGFR is overexpressed in a large number of tumours of epithelial origin, such as in 15–30% of breast cancers, 60% of lung cancers and 25–77% of CRC [113]. A considerable proportion of tumors developing on the left side of the colon overexpress EGFR, and this is associated with oncogenesis, progression and an increased risk of metastasis [114]. EGFR targeting-therapies have been described in metastatic CRC using monoclonal antibodies in different clinical assays [115].

Two monoclonal antibodies targeting the EGFR extracellular domain have reached the clinic, blocking EGFR signalling. Cetuximab is a chimeric monoclonal IgG1 mAb that binds to the extracellular domain of EGFR, causing its internalisation and degradation, while panitumumab is a fully humanised mAb [116,117]. However, these targeted therapies, which increase progression-free survival and overall survival, are effective only in the absence of downstream activating mutations in EGFR signalling, such as KRAS and NRAS [118]. In fact, right-sided colon cancers generally do not benefit from anti-EGFR therapy compared to left-sided tumours, due to their different embryological origin, as well as a higher number of mutations for KRAS [119]. 

## 6. Antibody-Based Immunotoxins in CRC Clinical Trials

The first toxins used for ITX design were mostly ricin and PE. Therefore, it is not surprising that all ITX entering CRC clinical trials up to date have been based on one of them (Table 1), despite the wide variety of toxins that are currently tested in preclinical settings.

### 6.1. Ricin-Based Immunotoxins

#### 6.1.1. Ricin-Based ITX Targeting p72

791T/36-RTA (XomaZyme-791) is composed of the murine mAb 791/36, obtained from animals immunized with the 791T sarcoma cell line, conjugated via a disulphide bond to ricin toxin A chain (RTA). The 791T/36 antibody binds to a tumor-associated antigen (gp72) expressed on a number of human tumors, including CRC and ovarian carcinomas in addition to osteogenic sarcoma. After encouraging preclinical results, a Phase I dose-escalation study was carried out in 17 mCRC patients. Biological activity, assessed as mixed tumor regression, was seen in five patients. Humoral antibody responses to the murine mAb and RTA components of the ITX were observed in all but one patient, including one who received a single injection of ITX [120]. In another phase I trial in patients with mCRC, dose-limiting toxicity was associated with VLS. Overall, no signs of objective clinical responses were observed in the 12 patients treated, and no further trials were conducted [121].

#### 6.1.2. Ricin-Based ITX Targeting CEA

The anti-CEA-bR ITX consists of the murine mAb I-1, which targets CEA, conjugated to ricin via a disulphide bridge. The whole intact toxin was used, with the ricin B-chain “blocked” to avoid nonspecific binding. A phase I/II study of the intralesional administration of anti-CEA-bR was conducted on 27 patients with mCRC [122]. To overcome the potential limitation of reduced antibody localization in tumour tissue, ITX administration was intralesional. In this trial, minor responses were seen in seven patients, but no major objective responses or changes in the growth rate of treated lesions were reported. Despite local administration, human anti-mouse IgG and anti-ricin antibody responses were observed. In this case, the trial was stopped before the development of dose-limiting toxicity, based on the difficulty to manufacture sufficient amounts of ITX in an academic setting and the failure to demonstrate any initial benefits.

### 6.2. PE-Based Immunotoxins

#### 6.2.1. PE-Based ITX Targeting Anti-Lewis Y

LMB-1 comprises the murine anti-Lewis Y mAb B3 chemically linked to PE38. In a pioneering study, LMB-1 was the first ITX to demonstrate a therapeutic effect in patients with solid/epithelial tumors after two decades of clinical trials [123]. In this phase I trial, from a total of 26 CRC patients, a 75% tumor reduction and resolution of clinical symptoms was observed in one CRC patient, and two other patients had minor responses. Nearly 90% of the patients developed antibodies against B3, and 100% against PE38. The major side effect of LMB-1 was VLS, manifested by hypoalbuminemia, fluid retention and peripheral oedema.

Subsequently, a phase II clinical trial (NCT00001805) studied the suppression of human anti-mouse (HAMA) and anti-toxin antibodies (HATA) to LMB-1 by the anti-CD20 mAb rituximab in patients with advanced carcinoma expressing the Lewis Y antigen. The development of LMB-1 was not pursued; instead, recombinant B3-based ITX targeting the Lewis Y antigen with potentially increased tumor penetration were designed and evaluated in clinical trials. LMB-7 (B3(Fv))-PE38 is a recombinant counterpart of LMB-1 with a molecular weight of 65 kDa, comprising the B3 scFv genetically linked to PE38. LMB-9 is composed of the disulfide-stabilized Fv (dsFv) of B3 fused to PE38. LMB-9 was tested in two phase I trials conducted on patients with advanced carcinomas. Renal toxicity was dose-limiting, probably due to the rapid clearance of the recombinant ITX by the kidney and their binding to Lewis Y antigen present in renal tubular cells. No significant antitumor activity was observed and no further data were published [16]. 

SGN-10 (BR96 sFv-PE40) is a recombinant, single-chain ITX composed of an scFv obtained from the anti-Lewis-Y BR96 mAb and the PE40 defective form of PE. In a clinical trial with 20 mCRC patients out of 46, dose-limiting events were due to gastrointestinal toxicity rather than to VLS. No complete or partial tumor responses were observed at an 8-week evaluation, although 31% of patients had stable diseases. The immunogenicity of the toxin moiety limited the bioavailability of SGN-10 by day 11 of therapy [124]. 

ScFv (FRP5)-ETA was constructed from the scFv of the humanized anti-HER2 FRP5 mAb genetically fused to PE38. A phase I trial, reported in 2003, was conducted on 11 patients with metastatic breast cancer and CRC (one patient) and malignant melanomas. The treatment was given by intra-tumor injection. Out of 11 patients, four showed complete remission and three showed partial remissions, although the CRC patient did not exhibit a significant response [129]. Another phase I trial was reported in 2005. This trial was conducted on 18 patients suffering from erbB2-expressing metastatic breast cancers, prostate cancers, head and neck cancers, non-small cell lung cancers (SCLC), or transitional cell carcinoma. In this trial, no objective responses were observed [130]. 

#### 6.2.2. PE-Based ITX Targeting EpCAM

The ITX MOC31PE consists of the murine anti-EpCAM mAb MOC31 covalently linked to the complete *Pseudomonas* exotoxin A (PE). After promising results in vivo [131], a phase I clinical trial of patients with metastatic CRC compared i.v. administration of MOC31PE alone, or in combination with cyclosporine A (CsA) to delay the emergence of anti-MOC31PE antibodies [125]. Regarding the safety profile, only a reversible increase in transaminases as dose-limiting liver toxicity was observed. The OS for the combination group was similar to that of patients on the last line of standard chemotherapy. Unexpectedly, patients treated with MOC31PE alone had a more prolonged overall survival (OS) time (median OS of 16.3 months vs. 6.0 months), although no radiological response according to RECIST criteria was assessed initially. The cytokine profile in patient sera and in vitro immunological studies suggested the induction of immunogenic death by MOC31PE leading to T-cell activation, suppressed in patients treated with CsA [126]. Indeed, HCT116 and SW480 CRC cell lines released HMGB1 when treated with MOC31PE, and the conditioned medium promoted DC maturation. Further studies should validate the potential of this ITX. 

A concern associated with most TAA is their expression in normal tissues, despite it being at lower levels, and the risk of on-target/off-tumor side effects, local administration being an alternative to avoid them. MOC31PE was also evaluated in a phase 1 trial of CRC patients with peritoneal metastasis as intraperitoneal (i.p.) treatment [128]. As expected, i.p. administration of MOC31PE was safe and well-tolerated, without signs of hepatotoxicity. It correlated with minimal systemic uptake, most likely due to its large molecular size of (>220 kDa) limiting the rate of drug absorption from the peritoneal cavity. However, all patients developed neutralizing antibodies. On the other hand, the large molecular size of MOC31PE could have impaired the tumor penetration when administered systemically in the previous trial.

#### 6.2.3. PE-Based ITX Targeting Muc-1

The ITX BM7PE is composed of the murine anti-Muc-1 mAb BM7 covalently linked to PE. BM7PE has demonstrated an ability to increase survival in experimental models, alone [132] or in combination with CsA [131]. This ITX is going to be tested in a phase 1–2 clinical trial, whose status as of October 2021 is “recruiting”. This phase 1/2 study will evaluate the safety, tolerance and dose of BM7PE in patients with CRC who have progressed to standard therapy or are intolerant to it. 

## 7. Antibody Fragment-Based Immunotoxins in CRC: Preclinical Developments 

### 7.1. PE-Based Immunotoxins

#### 7.1.1. PE-Based ITX Targeting CEA

The ITX hMN14(Fv)-PE40 is derived from a humanized anti-CEA scFv and the truncated PE40 [133]. The 15-aminoacid linker was modified to (GGSGS)_3_ instead of the canonical (GGGGS)_3_ to increase solubility. In cytotoxicity assays, the ITX showed specific growth suppression of CEA-expressing colon cancer cell lines MIP-CEA and LS174T. These IC50s correlated inversely with the surface expression of CEA, such that 50% killing was equivalent for each cell type when expressed in toxin molecules bound/cell (3000–5000). Interestingly, the presence of soluble CEA up to 1000 ng/mL did not affect the cytotoxicity against CEA-expressing cells (normal serum levels, <5 ng/mL).

#### 7.1.2. PE-Based ITX Targeting CD71 (Transferrin Receptor 1)

The transferrin receptor 1 (TfR1) levels on the cell surface, which modulate the uptake of TF binding iron, are associated with cell proliferation rate and, not surprisingly, are increased in tumor cells, including CRC cells. HB21-PE40 is an ITX made by fusing an scFv directed at TfR1 with the truncated PE40 [134]. Treatment of mice with HB21-PE40 only delayed the growth of s.c. tumors induced by CRC KM12L4 cells but eliminated KM12L4 liver metastasis. Moreover, the combination of HB21-PE40 with a proapoptotic, BH3-only mimetic, resulted in a substantial increase in anti-tumor activity compared with either treatment alone [135]. 

#### 7.1.3. PE-Based ITX Targeting Mesothelin

Mesothelin (MSLN) is a cell surface glycoprotein expressed at a high level on many malignancies including pancreatic adenocarcinoma, stomach cancer, ovarian cancer and epithelioid mesothelioma. An ITX named SS1P was constructed by fusing the Fv portion of an anti-mesothelin antibody to PE38. SS1P displayed a favorable safety profile in mesothelioma patients, but efficacy was limited by the development of neutralizing antibodies. LMB-100 is an improved, low-immunogenic version of SS1P, consisting of a humanized anti-mesothelin Fab and a PE24 mutated into domain III to remove B cell epitopes. LMB-100 is currently in Phase I/II trials in patients with mesothelioma, cholangiocarcinoma and pancreatic adenocarcinoma; however, the potential of mesothelin-targeted ITX in CRC has been overlooked. In a recent study, the sensitivity of CRC lines to LMB-100 in vitro was found to be similar to that of pancreatic adenocarcinoma cells [136]. Moreover, there was a significant delay in tumor growth in mice bearing CRC xenografts treated with LMB-100 as monotherapy and 50% complete regression in mice receiving combination treatment with Actinomycin D.

#### 7.1.4. PE-Based ITX Targeting CD25

Recently, it has been reported that an ITX kills mouse CD25+ cells, composed of 2E4 scFv fused to PE38 [137]. Obviously, CD25 is not a CRC TAA but a Treg marker, and the mechanism of action of this ITX is unique among all those commented above. Tregs (FOXP3^+^, CD25^+^, CD4^+^) contribute to the development of an immunosuppressive tumor microenvironment, inhibiting the activation and expansion of tumor-specific effector T cells. Intra-tumor administration of 2E4-PE38 in mice bearing one CRC tumor in each flank caused the regression of 100% of injected tumors and 92% of non-injected ones. Both treated and untreated tumors showed decreased numbers of Tregs and increased expression of effector CD8 T cell activation markers. Similar results were obtained in mesothelioma and breast cancer models. Moreover, all cured mice became resistant to rechallenge >50 days after tumor regression, showing that the treatment with 2E4-PE38 and subsequent depletion of Treg could induce long-term immunological memory.

### 7.2. α-Sarcin-Based Immunotoxins

Different first-generation sarcin-based ITX have been previously described, such as HB21 targeting the human transferrin receptor (TFR) [138] or conjugated to mAb-Fib-75 [139]. Despite their high specificity and efficacy in vitro, no further assays were performed, possibly due to the large size of these constructs obtained by chemical conjugation with the full-length antibody. Afterwards, the in vitro efficacy of a recombinant anti-TFR (scFv) ITX based on restrictocin was characterized [140]. More recently, new recombinant ITX designs based on sarcin against CRC, using different formats and targets, have been described and are summarised below.

#### 7.2.1. α-Sarcin-Based ITX Targeting GPA33

The recombinant ITX IMTXA33αS was obtained using humanised scFvA33, which showed high cytotoxic efficacy against different GPA33+ CRC lines, such as SW1222 and LIM1215 [141]. Furthermore, in vivo assays showed a significant reduction in tumor growth, angiogenic development and proliferative capacity of CRC xenografts in mice, providing a proof-of-concept of the potential therapeutic use of this ITX against CRC [142]. Moreover, comparative studies by confocal microscopy co-localisation assays including two other constructs, scFvA33T1 [143], based on RNase T1, and IMTXA33HtAΔ3W, based on a variant of the ribotoxin hirsutelin A, confirmed the relationship between the intracellular pathway followed by the toxins and their cytotoxic effectiveness [144]. Thus, they demonstrated that IMTXA33αS is internalized after binding to GPA33, mainly following a retrograde pathway through endosomes and the Golgi apparatus, with the release of α-sarcin to the cytosol by direct translocation due to the interaction between this ribotoxin and endosomal membranes [144]. 

Taking this into account, ITX designs including linkers with a specific furin cleavage site have been shown to be more cytotoxic [140]. These results confirm that intracellular processing and efficient release of the toxic domain to the cytosol are crucial for ITX design. In this line, two new CRC antitumor immunoRNases including a furin cleavage site were described, based in α-sarcin and RNaseT1, IMTXA33furαS and scFvA33furT1, respectively. The results confirmed not only the different intracellular routes followed by α-sarcin and RNaseT1 but also that both furin-variant designs exhibited significantly enhanced antitumor effectiveness compared to the parental ones [145]. Moreover, in vivo assays with IMTXA33furαS demonstrated their increased antitumor activity (Narbona J., Lacadena J., personal communication). 

#### 7.2.2. α-Sarcin-Based ITX Targeting CEA

The production and characterization of two new recombinant ITX targeting CEA have recently been described. IMTXCEAαS, exhibiting the classic ITX monomeric format, comprises the high-affinity anti-CEA scFv MFE23, obtained using phage display technology [146,147,148], fused to α-sarcin. On the other hand, IMTXTRICEAαS displays a trimeric conformation in solution [149]. This ITX was designed based on the trimerbody format of MEF23 scFv [150] which includes the trimerization domain from human collagen XVIII flanked by the scFv and the toxin [151]. Although both ITX exhibited antitumor activity, the trimeric ITX showed a highly improved therapeutic effect in comparison to its monomeric counterpart, both in vivo and in vitro. Indeed, trimeric ITX are endowed with increased functional avidity and exhibit enhanced properties, including longer half-lives and improved tumor targeting capacity. The characterization of the first ITX with trimeric format represents a step forward to the development of next-generation ITX [149]. New ITX designs targeting EGFR, based on VHH (VHHEGFRαS), are currently being characterized in both monomeric and trimeric formats (Narbona, J, et al. unpublished results).

### 7.3. Granulysin-Based Immunotoxins

#### 7.3.1. Granulysin-Based ITX Targeting CEA 

Intra-tumor injection of granulysin, although effective, would be difficult to apply in a clinical setting. Hence, an ITX comprising granulysin and the anti-CEA scFv MFE23 was generated to allow a systemic treatment [28]. The ITX formed by the fusion of MFE23 and granulysin (MFE23GRNLY) was tested in an in vivo preclinical model to demonstrate the targeted delivery of granulysin to CEA-expressing tumors. Previously, it was demonstrated in vitro that the ITX retained its specific binding to CEA and increased the cytotoxicity against several CEA-expressing tumors compared with granulysin alone. Most importantly, the systemic administration of MFE23GRNLY showed remarkable inhibition of tumor growth in mice xenotransplanted with CEA^+^ cells. This effect was not observed after systemic administration of granulysin alone, demonstrating the efficient targeting of the toxin mediated by the scFv moiety. The therapeutic effect was associated with decreased cellularity, apoptosis induction and NK cell infiltration in the tumor mass, suggesting a possible immunogenic effect [28]. The observed increase in cytotoxicity of the granulysin-based ITX can be explained by the concentration of a higher amount of granulysin molecules at the plasma membrane due to the binding to the antigen by the scFv moiety, in a process similar to the described “capping” of some antigens induced by antibodies [152].

#### 7.3.2. Granulysin-Based ITX Targeting the Tn Antigen

Another granulysin-based ITX directed against the Tn antigen has been developed, combining granulysin with an scFv derived from the anti-Tn mAb SM3. In principle, ITX directed against the Tn antigen would have a broader application, since it is overexpressed in a large variety of tumors [90]. In that study, an innovative procedure based on pulsed electric field technology was used to improve the yield of production of the ITX from yeast. It was demonstrated that SM3GRNLY retained its specific binding to the Tn antigen and increased the cytotoxicity against these Tn^+^ cell lines compared with granulysin alone [27]. However, no in vivo studies with this ITX have been reported yet.

### 7.4. Cholera Exotoxin-Based Immunotoxin

HB21-CET40 comprises the above-commented anti-TfR1 scFv HB21 and amino acids 270 to 634 (domains II and III) of cholera exotoxin (CET) from *Vibrio cholerae*, structurally similar to PE from *Pseudomonas*. HB21-CET40 was assayed for cell-killing activity against several cell lines including the CRC DLD-1, being ten times less active when compared to HB21-PE40 [153]. The potential of using both ITX sequentially was suggested, once patients developed an immune response to PE40. 

### 7.5. Cucurmosin-Based Immunotoxin

The anti-EGFR single domain (sdAb) 7D12-based recombinant ITX rE/CUS incorporated cucurmosin (CUS) from *Cucurbita moschata* as toxic moiety [154]. In further development, a second anti-EGFR sdAb (9G8), which does not compete for binding to EGFR with the first one, was added to the construct to produce Bs/CUS. Although “Bs” stands for bispecific, perhaps it would be more appropriate to use the denomination of biparatopic. In cytotoxicity assays with the CRC cell line SW1116, Bs/CUS had a 55-fold increase in activity at 72 h compared to rE/CUS [155]. Given the small size of each sdAb, the molecular weight of Bs/CUS (58 kDa) is still under the glomerular filtration threshold. For this reason, fusion to an albumin-binding domain was proposed in order to extend the half-life before pursuing in vivo experiments. The pre-clinical studies mentioned in this section and some additional ones are summarized in Table 2.

## 8. Challenges and Future Directions in ITX Design

### 8.1. Immunogenicity of the Toxin Portion

Humanized and fully human antibodies are currently available as targeting moieties; however, most toxins are still highly immunogenic. This is a great limitation for non-human toxins, which clearly constrains their clinical success [22]. In patients with hematological malignancies, the formation of neutralizing antibodies against foreign toxins is low, probably due to the fact that their immune system is impaired by previous chemotherapy and the nature of their condition. However, in patients with solid tumors such as CRC, the rate of antibody formation is much higher and this restricts ITX administration to one or two cycles [122].

The design of fourth-generation ITX, in which human cytotoxic proteins replace the foreign toxins, has been described in Section 4. ITX conjugation to high-molecular-weight polyethylene glycol has been used to attenuate their immunogenicity; however, pegylation of ITX might also reduce their clinical efficacy. Another strategy is the administration of the ITX along with immunosuppressive agents, with T-cell and/or B-cell depleting chemotherapies or with mAb, such as the anti-CD20 rituximab, to minimize anti-toxin antibody responses [6]. Alternatively, mutagenic de-immunization of non-human toxins has been proposed [164,165]. This approach has been systematically pursued by the Ira Pastan group, which has modified the PE toxin in order to eliminate epitopes recognized by both neutralizing antibodies and by T cells [166,167], generating, for example, the improved mesothelin-targeting LMB-T14 ITX [164]. Curiously, the elimination of a conformational B-cell epitope created a new T-cell epitope in the toxin, indicating the complexity in the interaction between the immune system and toxins [168]. Another group has also mutated the P38 moiety in a trastuzumab-based ITX with good preclinical results [169]. 

### 8.2. Endosomal Retention of Toxins

Several approaches to solve the endosomal retention of toxins after ITX uptake have been developed, comprehensively reviewed by Fuchs et al. [170]. Bostad et al. used a CD133-directed, saporin-based ITX combined with a photosensitizer. The light treatment produced high endosomal ROS production, leading to their rupture and toxin release to the cytoplasm. This treatment was tested in vivo in a murine model xenotransplanted with the CRC cell line WiDr, exposing tumors to a 652 nm laser [159]. The activity of ITXs directed against the glycoprotein A33 conjugated with bacterial or fungal toxins, such as the ribotoxin α-sarcin or the RNase T1, have been improved by the inclusion of a furin cleavage site, allowing endosomal cleavage of the ITXs and a more efficient liberation of the toxins to improve their therapeutic activity [145]. Simultaneous treatment with saponins increases the endolysosomal escape of ITX-associated toxins, such as saporins, for which membrane cholesterol seems to be necessary [171]. 

### 8.3. Short Half–Life

Classical strategies to increase the half-life of small proteins include modification with polyethylene glycol or fusion to an Fc region or serum albumin. As an alternative, ITX containing albumin-binding domains (ABD) have been reported that considerably increase their half-life in the circulation and antitumor activity in mice [172]. ITX comprising an ABD from *Streptococcus*, or an anti-albumin single-domain antibody, showed half-lives in the circulation of mice ranging from 113 to 194 min, compared with 13 min for an ITX with no ABD. Moreover, ABD–ITX fusion proteins exhibited enhanced therapeutic effects in nude mice bearing human pancreatic carcinoma xenografts.

### 8.4. Bispecific Immunotoxins

Multimerization is another strategy that may allow the increase of ITX size above the renal threshold, improving the half-life as well as optimizing tumor specificity and decreasing on-target off-tumor side effects [173]. It has been proposed that a dual-targeted anti-tumor BsAb recognizing two different TAA should preferentially bind to malignant cells if the affinities of the individual binding domains are sufficiently low as to require the presence of both target antigens for efficient binding, due to the avidity effect, whereas high-affinity binding domains may also bind normal cells expressing only a single TAA [174]. In addition, dual targeting of two TAA may contribute to preventing the tumor escape by antigen loss caused by selective pressures from conventional single TAA-targeting ITX and may help to overcome preexistent antigenic heterogeneity. The BsITX DTEpCAM23 (diphtheria toxin fused to two scFv anti-EpCAM and anti-HER2) was assayed in vitro against the HT-29 human CRC cell line, which expresses medium levels of HER2 and high levels of EpCAM. Interestingly, whereas the anti-HER2 monospecific ITX was not able to affect the proliferation of the HT-29 cell, the bispecific ITX showed a 10-fold increase in activity over the monospecific anti-EpCAM ITX [175]. Moreover, i.p. administration of the bispecific ITX showed enhanced efficacy in mice bearing HT-29 xenografts, along with diminished toxic effects, when compared to monospecific ITX. In a recent study, a PE38-based anti-HER2 x anti-PDGFRβ BsITX incorporating an IgG-binding domain for increased half-life was described. This BsITX exhibited improved efficacy against a gastric carcinoma xenograft model [176]. Another strategy under development aimed at CRC treatment is an α-sarcin-based bsITX simultaneously targeting EGFR and GPA33 on the tumor cells (Narbona, J, et al. unpublished results).

### 8.5. Gene Therapy for Immunotoxin In Vivo Production

An alternative to the administration of purified protein is the use of gene-based strategies for in vivo production of antibody-based therapeutics [177]. This strategy could compensate for the rapid blood clearance of recombinant antibody fragment-based ITX and circumvent the challenges associated with large-scale production and high costs. This goal can be accomplished by ex vivo genetically modified cells [178,179] or direct in vivo delivery of encoding sequences. The use of T lymphocytes as a cell delivery system is appealing for cancer treatment, given their capacity of tumor localization when adoptively transferred [180]. Transduction with viral vectors is an efficient method for gene delivery, but it has associated risks. Transfection with mRNA has a better safety profile. Recently, it was reported that human primary T cells transfected with mRNA encoding and the fusion protein anti-HER2 scFv-PE38 were able to secrete the ITX, which had cytotoxic activity toward HER2+ carcinoma cells in vitro [181].

In recent years, there has been growing interest in modified mRNA-based in vivo gene delivery for different therapeutic applications because of its advantages over classical viral and non-viral vectors [182,183]. Indeed, several antibody fragments expressed after i.v. infusion of encoding mRNA are in preclinical studies [184] and the first phase I clinical trial of an mRNA-encoded antibody has recently been completed (NCT03829384). A similar approach could be used for the expression of small third-generation ITX, improving pharmacokinetics and achieving more cost-effective production and a faster translation to clinical evaluation.

## 9. Conclusions

ITX constitute an appealing approach for CRC therapy, with a variety of targets and toxins tested in the last 30 years. Pioneering ITX clinical assays in CRC were not successful but paved the way for the design of new versions of ITX with improved properties currently under development. Some challenges related mainly to pharmacokinetics, cytosolic delivery and especially with the immunogenicity of non-human toxins remain. To fullfil the unmet need for new treatments in advanced CRC, these issues should be addressed. Promising strategies for deimmunization, improved endolysosomal escape and extended serum half-life are being actively investigated.

## Figures and Tables

**Table 1:** Immunotoxins tested in colorectal cancer patients.

ITX	Toxin	Antibody Format	Target	Clinical Trial	Phase	Ref.
Xomazyme-791	Ricin	mo IgG2b	72kDa	N/A	Phase 1	[120,121]
Anti-CEA-bR	Blocked ricin	mo IgG	CEA	N/A	Phase 1/2	[122]
LMB-1	PE38	mo IgG1	Lewis Y	NCT00001805	Phase 2	[123]
LMB-9	PE38	dsFv	Lewis Y	NCT00019435 NCT00005858	Phase 1 Phase 1	[16]
SGN-10	PE40	scFv	Lewis Y	N/A	Phase 1	[124]
MOC31PE	PE	mo IgG1	EpCAM (CD326)	NCT01061645 NCT02219893	Phase 1 Phase 1/2	[125,126,127,128]
BM7PE	PE	mo IgG1	MUC-1	NCT04550897	Phase 1/2	N/A

Abbreviatons: CEA, carcinoembryonic antigen; dsFv, disulfide-stabilized Fv; EpCAM, epithelial cell adhesion molecule; MUC-1, mucin 1; mo, mouse; N/A, not available; PE, *Pseudomonas* exotoxin; scFv, single-chain Fv.

**Table 2:** Immunotoxins in preclinical development for CRC treatment.

ITX	Toxin	Antibody Format	Target	Ref.
2E4-PE38	PE38	scFv	CD25	[137]
SWA11-ZZ-PE38	PE38	mo IgG2a	CD24	[156]
HB21-PE40	PE40	scFv	TFR	[134,135,157]
LMB-12 LMB-100 LMB-164	PE24 PE24 * PE24	dsFv Hz Fab dsFv+ABD	Mesothelin	[136]
hMN14(Fv)-PE40	PE40	Hz scFv	CEA	[133]
HB21-CET40	CET40	scFv	TFR	[153]
3-17I-saporin	saporin	hu IgG1	EpCAM (CD326)	[158]
AC133-saporin	saporin	mo IgG1	CD133	[159]
DTEpCAM23	DT	(scFv)_2_	EpCAM x HER2	[160]
MFE23-GRNLY	granulysin	scFv	CEA	[28]
SM3GRNLY	granulysin	scFv	Tn	[27]
IMTXA33αS IMTXA33furαS	α-sarcinE	scFv	GPA33	[141,144,145]
IMTXCEAαS IMTXTRICEAαS	α-sarcin	scFv, trimerbody	CEA	[149]
scFvA33T1 scFvA33furT1	RNase T1	scFv	GPA33	[144,145]
IMTXA33HtA3ΔW	hirsutellin A	scFv	GPA33	[161]
T84.66-gelonin	gelonin	mo IgG1	CEA	[162]
MAbC27-abrin A	abrin A	mo IgG1	CEA	[163]
Bs/CUS	cucurmosin	bp (sdAb)2	EGFR	[155]

Abbreviatons: ABD, albumin binding domain; bp, biparatopic; CEA, carcinoembryonic antigen; CET, cholera exotoxin; DT, diphtheria toxin; dsFv, disulfide-stabilized Fv; EGFR, epidermal growth factor receptor; EpCAM, epithelial cell adhesion molecule; Fab, antigen-binding fragment; mo, mouse; hz, humanized; hu, human; PE, *Pseudomonas* exotoxin; scFv, single chain Fv; sdAb, single domain antibody; TFR, transferrin receptor; *, B-cell epitope-removed.

## Data Availability

Not applicable.
